# Brown adipocyte-derived exosomes in type 2 diabetes mellitus impair endothelial function via regulating intracellular calcium cycle

**DOI:** 10.3389/fcvm.2025.1546325

**Published:** 2025-05-09

**Authors:** Xiaojie Ruan, Wei Zhao

**Affiliations:** ^1^ Ningxia Institute of Clinical Medicine, People’s Hospital of Ningxia Hui Autonomous Region, Ningxia Medical University, Yinchuan, China; ^2^ Department of Biochemistry and Molecular Biology, Basic Medical College, Ningxia Medical University, Yinchuan, China

**Keywords:** type 2 diabetes mellitus, endothelial dysfunction, atherosclerosis, brown adipose tissue, exosomes

## Abstract

**Background:**

Atherosclerosis is a leading cause of macrovascular complications in type 2 diabetes mellitus (T2DM). Lipid metabolism disorders in T2DM alter exosomal cargos, affecting vascular endothelial cells and impairing vascular endothelium-dependent relaxation.

**Objective:**

This study investigates the link between T2DM and atherosclerosis, focusing on adipose tissue-derived exosomes (AT-Exosomes) as key pathogenic factors in T2DM.

**Methods:**

AT-exosomes derived from diabetic (C57BLKS-Lepr^db/db^) and non-diabetic (C57BLKS-Lepr^db/+^) mice were co-cultured with vascular aorta to evaluate pathogenicity. RNA screening in mouse aortic endothelial cells (MAECs) identified differential genes impacted by T2DM brown adipose tissue (BAT)-derived vs. healthy BAT-derived exosomes.

**Result:**

BAT-derived exosomes significantly disrupted endothelium function compared to white adipose tissue (WAT)-derived exosomes. Inositol 1,4,5-trisphosphate receptor type 3 (ITPR3) gene expression in MAECs was significantly reduced in diabetic mice. Functional studies revealed that ITPR3 positively regulates the Ca^2+^/CAMKII/eNOS signaling pathway to inhibit nitric oxide (NO) release, impairing endothelial relaxation.

**Conclusion:**

BAT-derived exosomes in T2DM reduce ITPR3 expression in endothelial cells, lowering intracellular Ca^2+^ and NO production, thereby contributing to vascular endothelium-dependent relaxation dysfunction. Targeting this pathway may offer therapeutic insights for T2DM-associated vascular complications.

## Introduction

Diabetes and its complications are a significant global health challenge, with far-reaching impacts on the cardiovascular system, nervous system, kidneys, retina, and liver. These complications notably increase the risk of adverse outcomes in affected individuals (1). Among these, atherosclerosis, a major cause of mortality worldwide, is exacerbated in type 2 diabetes mellitus (T2DM) due to lipid metabolic disorder and chronic inflammation. Although there are some substantial advances in understanding diabetic complications, the research on diabetes vascular aortic complications is still limited.

Atherosclerosis progression is influenced by adipose tissue metabolism. Recent studies have shown that adipose tissue-derived exosomes (AT-Exosomes) serve as significant intercellular communicators to target various organs through signaling mediated by surface receptors (2). AT-Exosomes play an important role in regulating key processes such as inflammation resolution, angiogenesis, and the remodeling of the extracellular matrix (3). Additionally, they are recognized for their protective effects on cardiomyocytes (4).

These small membrane-bound vesicles carry molecules including DNA, mRNA, and microRNAs, reflecting the physiological conditions under which they are released (5). Under metabolic homeostasis, AT-Exosomes have a function of tissue repair and regeneration (6–8). For instance, exosomes from adipose mesenchymal stem cells can protect endothelial cells from atherosclerosis via microRNA-342-5p and miR-221-3p (9, 10). However, exosome component can be changed by micro-environment *in vivo*, it has been showed that diabetic AT-exosomes reduced protective effects and heightened contributions to vascular dysfunction in diabetic mice (11). High glucose conditions further drive endothelial cell-derived exosomes to stimulate calcification and senescence of vascular smooth muscle cells, linking T2DM to atherosclerosis, periarterial sclerosis, and coronary heart disease (12). This highlights that exosomes can be investigated not only as potential therapeutic modalities for diseases but also as the key factors in disease progression. Due to the paracrine effects, exosomes are particularly applicable in studying disease complications, allowing for a deeper exploration of the pathogenic pathways involved. For instance, this includes examining the biological molecular mechanisms behind complications like cardiovascular disease in diabetes.

Adipose tissue can be broadly divided into white adipose tissue (WAT) and brown adipose tissue (BAT), with the latter being highly active in glucose and lipid metabolism (13). BAT plays a crucial role in maintaining glucose homeostasis, improving *β*-cell function, and reducing insulin demand (14). However, in T2DM, BAT exhibits pro-apoptosis properties, releasing inflammatory factors and exosomes that contribute to vascular damage (15, 16). Recent studies have shown that BAT-derived exosomes are potent regulators of immune and vascular endothelial cells under inflammatory conditions. Therefore, AT-exosomes can be studied as potential interaction vectors in vascular complications of diabetes.

This study investigates the role of AT-Exosomes, particularly from BAT, in vascular endothelial dysfunction in T2DM. By analyzing exosomes from diabetic and non-diabetic mice, we aim to uncover key factors underlying the endothelial damage in induced by BAT-derived exosomes, contributing to the development of atherosclerosis in diabetes.

## Materials and method

### Exosome preparation and identification

Male diabetic mice and non-diabetic mice (Cavens, China) aged 6–8 weeks were used to obtain brown adipose tissue from the interscapular region, intact epididymal white fat from the vicinity of the testis, intact perirenal white fat from the kidney and intact subcutaneous white adipose tissue from the subcutaneous area under aseptic conditions. 0.4 g of each adipose tissue was isolated for the tissue culture sample. Adipose tissue was cut into small pieces and cultured in the medium mixed with 1.8 ml Minimum Essential Medium (MEM Alpha 1X, Gibco, Gaithersberg, MD, USA) and 0.02 ml of the antibiotic penicillin-streptomycin (Pen Strep, Gibco, Gaithersberg, MD, USA) for 3 days at 37℃ and 5% CO_2_. The supernatant of the Petri dishes was collected after 12,000 × g 10 min centrifugation to remove tissue fragments. The supernatant was collected using the Ultra-15 concurrent filter unit (Amicon Ultra-15, Ireland), with a processing time of 30 minutes at 39,000 × g. The remaining filtered liquid was absorbed and collected for further centrifugation, undergoing a rigorous process at 100,000 × g for one hour after being filtered twice through a 0.22 μm filter at a temperature of 4℃ in cryocentrifuge (XPN-100, Beckman, USA). After that the Centrifugal sedimentation will be resuspended into 1 ml PBS. The samples were stored in a refrigerator at −80 ℃ for subsequent experiments.

The qualification of exosomes was performed using a transmission electron microscope (TEM, H-750, Hitachi, Japan). The size distribution of exosomes was measured by Nanoparticle tracking analysis (NTA) NS300 (Malvern, Britain). 0.05 ml of sample solution containing exosomes was used in the laminar flow purification covered with volume fixed to 1 ml in a 1.5 ml miniature centrifugation tube with PBS for preparation for the NTA. The exosome concentration is determined by the number of particles of 40 nm to 160 nm in size.

Exosome protein profiles were characterized by Western blot, with special attention to the presence of exosome markers such as HSP70, CD63, and CD81(Proteintech, USA)**.** 20 μl sample was injected into each well and 2 μl of standard dye (Yeasen, China) was injected into the first and final gel holes to locate the analysis. Imprinted blots of different molecular sizes were placed in TBST of 1:2000 dilutions of anti-rabbit CD63 antibody, TBST of 1:2,000 dilution of anti-rabbit HSP70 antibody, and TBST of 1:2,000 dilution of anti-rabbit CD81 antibody respectively, and incubated overnight in a dark room of 4℃. TBST of 1:2000 dilution of anti-rabbit Calnexin antibody (Zen-bioscience, China) was used as a protein marker in negative control samples. After being washed with TBST for 3 times, 10 min each time, the enzyme-conjugated secondary antibody (Zen-bioscience, China) was incubated with membrane for 1 h. Finally, the film was developed using the enhanced chemiluminescence detection system (ECL Reagent, Thermofisher) and exposed on x-ray film.

### Wire myograph detection

Balb/C mice aged 8-12 weeks were sacrificed with cervical dislocation. The heart and kidney were gently lifted with forceps and the tissue was transferred to an anatomical plate containing PBS solution for fixation.

The aorta was dissected under a microscope. The entire artery tissue was immersed in the solution. The fatty tissue around the artery was clamped with forceps and all connective tissue was cut with a dissecting shear to isolate the artery. The excess artery was clamped with forceps and the artery was transferred to a calcium-free Krebs-Henseleit cold solution. The artery was cut, and both sides of the artery segment were carefully opened with two forceps. The blood was gently dripped from the vessel, then the end of the incision was swollen with tweezers and the treated vessel was cultured. The medium was prepared by mixing 1.8 ml Dulbecco's Modified Eagle's Media (DMEM, Gibco, Gaithersberg, MD, USA), 0.02 ml of antibiotic penicillin-streptomycin (Pen Strep, Gibco, Gaithersberg, MD, USA), and 0.2 ml of extracted exosome solution (1,000 particles). After each vessel was placed in the medium, the samples were placed in a tissue incubator for two days.

Two lengths of 2.5 cm stainless steel wire were prepared and placed on the same plate. One end of the artery with a pair of forceps was gently clamped with forceps and the other forceps were used to insert the two threads, one at a time, into the lumen of the artery. The wires stayed flat didn't touch the endothelial cells. Two tweezers were used to clamp the sides of one of the wires and the blood vessel was placed in the space between the jaw to secure it. Endothelium-dependent relaxation (EDR) was determined by adding acetylcholine (ACh, 3 nmol/L–10 μmol/L) to phenylephrine (Phe, 3 μmol/L) in the pre-contraction segment. The endothelium-dependent relaxation test was performed with exogenous nitric oxide (NO) donor sodium nitroprusside (SNP, 1 nmol/L–10 μmol/L).

### Establishment of cell model

Model I: MAECs and human aortic endothelial cells (HAECs) were brought from Bluefbio Co., LTD in Shanghai. After MAECs and HAECs were grown to 60% density in 6-well plates, cells were cultured for 36 h after 150 μl of exosomes suspended with PBS were respectively added to the holes. Cells were then used to detect CamkII and eNOS protein phosphorylation, eNOS activity, and NO secretion concentration.

Model II: MAECs and HAECs were cultured to 60% confluence in a 6-well plate, followed by the addition of 250 μl siRNA (Riobio, China) and lipo2000 mixed liquid or 250 μl plasmid (Genechem, China) and lipo2000 (Thermo Fisher Scientific Limited, China) mixture into each well after 36 hours of culture. The cells were collected separately, and total RNA was extracted using a dedicated kit. After cDNA synthesis, qPCR assay was performed to evaluate the knockdown efficiency or overexpression efficiency of the ITPR3 gene in the cells. Western blot analysis will be conducted to assess changes in ITPR3 protein expression levels. The prepared samples will be loaded at a volume of 20μl per hole for electrophoresis and transferred onto membranes at 120 V for 1.5 h with a current of 220 mA. Subsequently, the membranes will be blocked with a solution containing 5% BSA (Bjbiotopped, China) for 40 min before incubation with ITPR3-rabbit antibody (ABclonal, China). Detection of secondary antibody binding will be carried out using rabbit-specific antibodies. Internal reference proteins will be probed using β-actin-rabbit antibody (Proteintech, USA) at a dilution ratio of 1:10,000.

### NO detection

NO assay kit(Nanjin Jiancheng Bioengineering Institue, China) was used to detect the amount of NO secreted by treated cells in the two cell models. The prepared cell suspension was centrifuged at 1,000 × g/min for 10 minutes, the supernatant was discarded, and the cells were left to precipitate. After precipitating the suspended cells with PBS, they were centrifuged at 1,000 × g/min for 10 min and cleaned once, leaving the cell precipitate and adding 200 μl PBS for re-suspension, the cells were broken with an ultrasonic breaker. 300 μl of homogenate supernatant was taken and the prepared mixed reagent was added, and the cells were left for 10 minutes, and centrifuged at 4,000 × g/min for 15 minutes. 160 μl supernatant and 80 μl color developer were taken and left for 15 min, and NO concentration was detected by 550 nm emission light using an enzyme label instrument (Varioskan LUX, Thermofisher, USA).

### eNOS activity analysis

Mouse endothelial nitric oxide synthase (eNOS) ELISA kits (Meilian, China) and human endothelial Nitric oxide synthase (eNOS) ELISA kits (Meilian, China) were used to detect changes in cell eNOS activity in two cell models. The absorbance (OD value) of each hole was measured at 450 nm wavelength to calculate eNOS activity.

### RNA extraction and gene expression analysis

The samples were frozen with dry ice and sent to Shanghai Shengyin Biological Co., LTD for RNA sequencing. Total RNA extraction and quantitative PCR (qPCR) were performed on the cell bodies from brown adipose-derived exosomes co-cultured with MAECs using RNA extraction kits (Bioteke, China). The total RNA obtained was synthesized into cDNA using a high-volume cDNA reverse transcription kit (TaKaRa, Shiga, Japan) and used as a template for primer group qPCR (Table 1). The relative mRNA expression level was normalized to β-actin by ΔCt method.

**Table 1:** Primer sequences used for qPCR analysis.

Gene name	Gene ID	Primers	
		Forward	Reverse
β-actin	11461	AGAGGGAAATCGTGCGTGAC	CCATACCCAAGAAGGAAGGCT
Klc3	232943	GCTCAACATCCTGGCACTCG	CCATAGAGGACAGCCAGGTTG
Itpr3	16440	GGGCGCAGAACAACGAGAT	GAAGTTTTGCAGGTCACGGTT
Radil	231858	GACTCTACATACAGACCCTGCT	CCTCCATGTCGGATCAAATCCA
Nkx1-1	672284	TTCAACAGTAGGAGACGCCG	TTCAACAGTAGGAGACGCCG
Gpr25	383563	CTGGGACTATTCGGGCTCG	AAGTGCAGCACGAAGGTGTC
Mib2	76580	CGTACAGTTGTCGTTCAGTGG	TTTGGGCGTTGTCATAGAGCA
Disp3	242748	CCGGAGGGTCAGGTAACCA	CTCACATAGGTGCGCGAGT
Ptpru	19273	GCTCAACATCCTGGCACTCG	CCATAGAGGACAGCCAGGTTG
Ddr1	12305	ATGCTGACATGAAGGGACATTT	GGTGTAGCCTACGAAGGTCCA
Tbx2	21385	CCGATGACTGCCGCTATAAGT	CCATCCACTGTTCCCCTGT

### Detection of phosphorylation levels of the CamkII and eNOS proteins

Western blot was used to detect the changes in the levels of phosphorylation of the CamkII and eNOS proteins in two-cell modes. After the prepared cell samples were sampled at a volume of 25 μl per space. Incubation is performed using p-CamkII-rabbit antibody (Zen-bioscience, China) and p-eNOS-Ser117-rabbit antibody (Zen-bioscience, China) after 40 min isolation in 5% BSA solution, and exposure detection is carried out after rabbit secondary antibody incubation (Zen-bioscience, China). The membrane regeneration solution is used for 15 min to remove the bound antibodies, and after incubation with CamkII-rabbit antibody (Zen-bioscience, China) and eNOS-rabbit antibody (Zen-bioscience, China), the total protein content of the incubated rabbit secondary antibody (Zen-bioscience, China) is detected by exposure. β-actin-rabbit antibody at 1:10,000 is used for incubation of internal reference proteins and quantified with Image J.

### Statistics analysis

Statistical analysis was performed using the GraphPad Prism 8.0.1 software. Each experiment was independently repeated three times. Data are expressed as average dry SD. The normal distribution of the data is confirmed by the normality test and log-normality test. One-way ANOVA was used to analyze the differences between multiple groups, and bidirectional ANOVA was used to analyze the differences between groups split into two independent variables. Brown-Forsythe and Welch's analysis of variance was used for correction analysis. A *t*-test is performed between two comparable groups. When the data distribution is abnormal, the nonparametric Kruskal–Wallis test is used to compare the medians. *P* < 0.05 was considered statistically significant.

## Result

### AT -exosomes were extracted and identified

**Figure 1:**
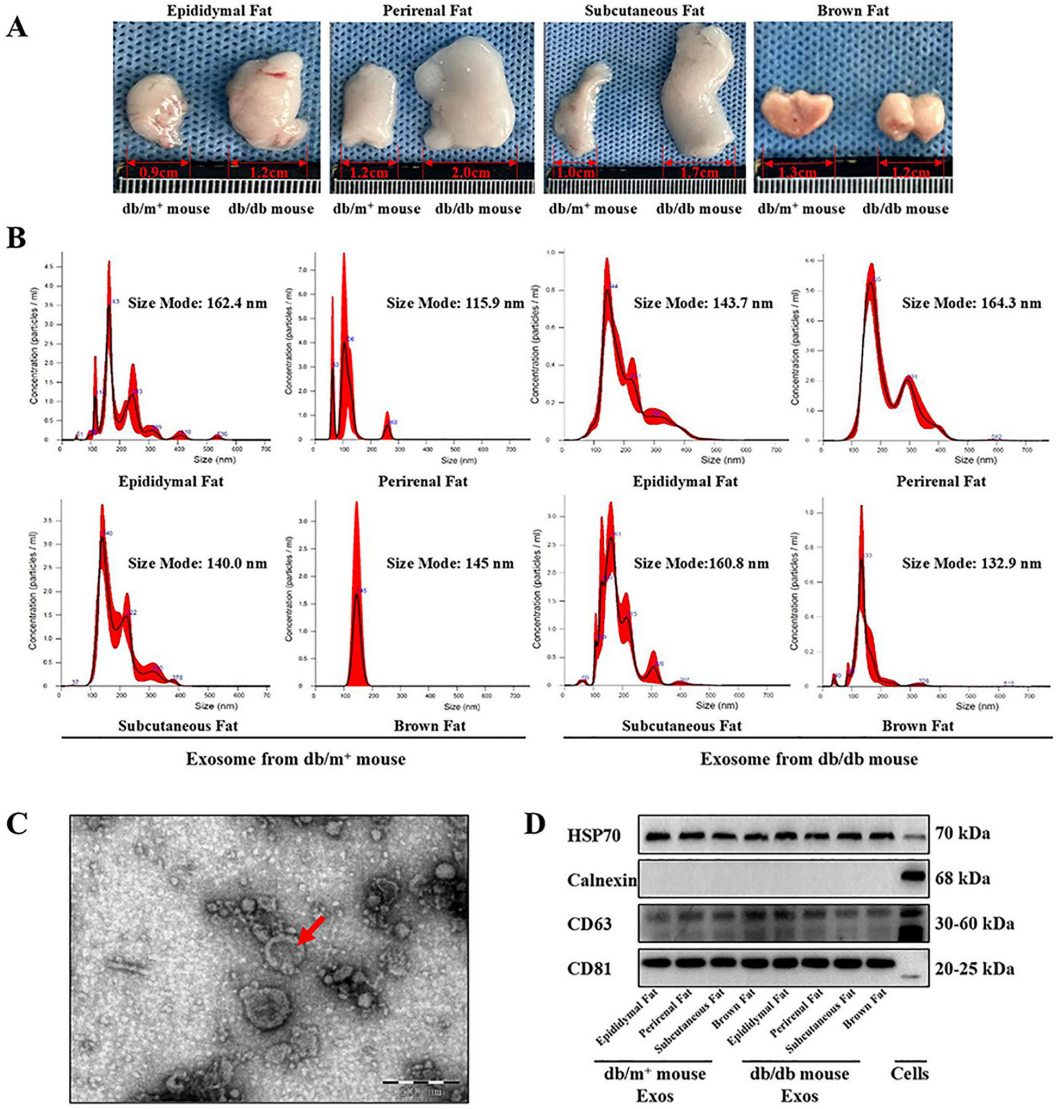
Extraction and identification of exosomes derived from adipose cells. **(A)** Epididymal fat, perirenal fat, subcutaneous fat and brown fat were clipped from diabetic mice and non-diabetic mice, and the culture medium supernatant was obtained for exosomes extraction. **(B)** The particle size of the lipogenic exosomes extracted in this experiment was detected by NTA experiment, and the particle size was judged to be between 115 nm and 165 nm, *n* = 3. **(C)** Particulate matter in the form of exosomes vesicles was detected from the suspension containing lipogenic exosomes by TEM observation, *n* = 3. **(D)** exosomes’ marker proteins were detected by western-blot. CD81, CD63 and HSP70 were detected as positive marker proteins of exosomes, *n* = 3.

Epididymal, perirenal, subcutaneous, and brown adipose tissues were harvested from diabetic and non-diabetic mice, and cultured for 72 h, and their exosomes were isolated from the supernatant via ultracentrifugation (Figure 1A). Nanoparticle tracking analysis (NTA) showed that the exosome diameters ranged from 115.9 nm to 164.3 nm (Figure 1B). The characteristic morphology of exosomes was observed under TEM (Figure 1C). Western blot analysis detected the positive marker proteins HSP70, CD63, and CD81 in the exosome samples, while the negative marker protein Calnexin was absent (Figure 1), indicating high exosome purity suitable for subsequent studies.

### BAT-derived exosomes influence vascular aorta’s vasorelaxation

**Figure 2:**
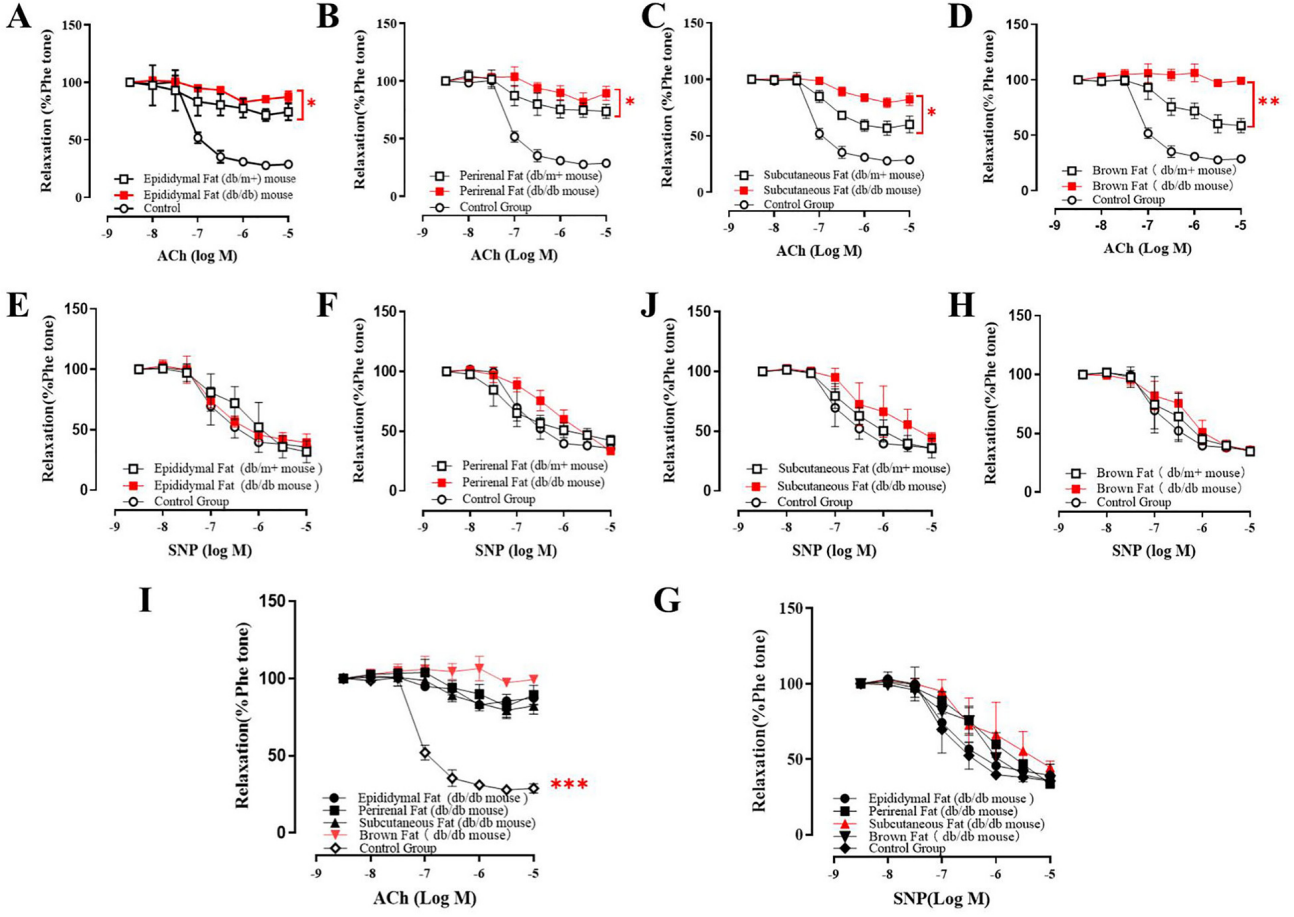
Co-culture of adipocyte derived exosomes and mice vascular aorta. **(A–D)** The vasorelaxation function of ACh treated mice co-cultured with different type of AT-exosomes from diabetic mice and non-diabetic mice. The differences were statistically significant between experiment groups, *p ≤ 0.05, **p ≤ 0.01, *n* = 3. **(E–H)** Effect of AT-exosomes from diabetic mice on vasorelaxation after the addition of SNP, compared with non-diabetic mice. **(I)** The difference of vasorelaxation in vascular aorta cocultured with AT-exosomes from different adipose tissue and PBS. ***p ≤ 0.001, *n* = 3. **(G)** After the addition of SNP, the vasorelaxation of vascular aorta co-cultured with AT-exosomes from different adipose tissue and PBS, *n* ≥ 3.

The vascular effects of AT-Exosomes were evaluated using isolated aortic segments from various adipose depots cultured *in vitro*. Endothelium-dependent relaxation (EDR) was determined by adding acetylcholine (ACh) and phenylephrine (Phe) to pre-constricted segments. eNOS activation, induced by shear stress from blood flow or chemical stimuli such as Ach, catalyzes the conversion of L-arginine to NO, which diffuses into vascular smooth muscle cells, leading to relaxation (17, 18). To differentiate the effects of exosomes on vascular endothelial vs. smooth muscle cells, perirenal and subcutaneous adipose-derived exosomes from diabetic mice were applied to these segments. The results showed that both perirenal and subcutaneous AT-derived exosomes from diabetic mice significantly impaired aortic vasodilation compared to exosomes from non-diabetic mice (Figures 2A–D). The relaxation dysfunction caused by BAT-derived exosomes was more significant in diabetic mice than in controls (Figure 2D). An endothelium-independent relaxation test, using the exogenous NO donor sodium nitroprusside (SNP) showed that all exosomes caused impaired vasodilation. However, supplementation with SNP restored relaxation in all samples, indicating that the exosomes from diabetic mice impaired vasodilation by affecting aortic endothelial cell function, rather than smooth muscle activity (Figures 2E,F,J,H). Notably, BAT-derived exosomes from diabetic mice caused more significant relaxation dysfunction compared to other adipose-derived exosomes (Figures 2I,G).

### Diabetic AT-derived exosomes reduce NO secretion via inhibiting eNOS phosphorylation

AT-derived exosomes from diabetic mice were found to impair vascular endothelium-dependent vasodilation, with endothelial eNOS identified as the key enzyme regulating NO production and vasodilation. To validate these findings, we co-cultured exosomes from different adipose tissue depots with MAECs and HAECs respectively.

**Figure 3:**
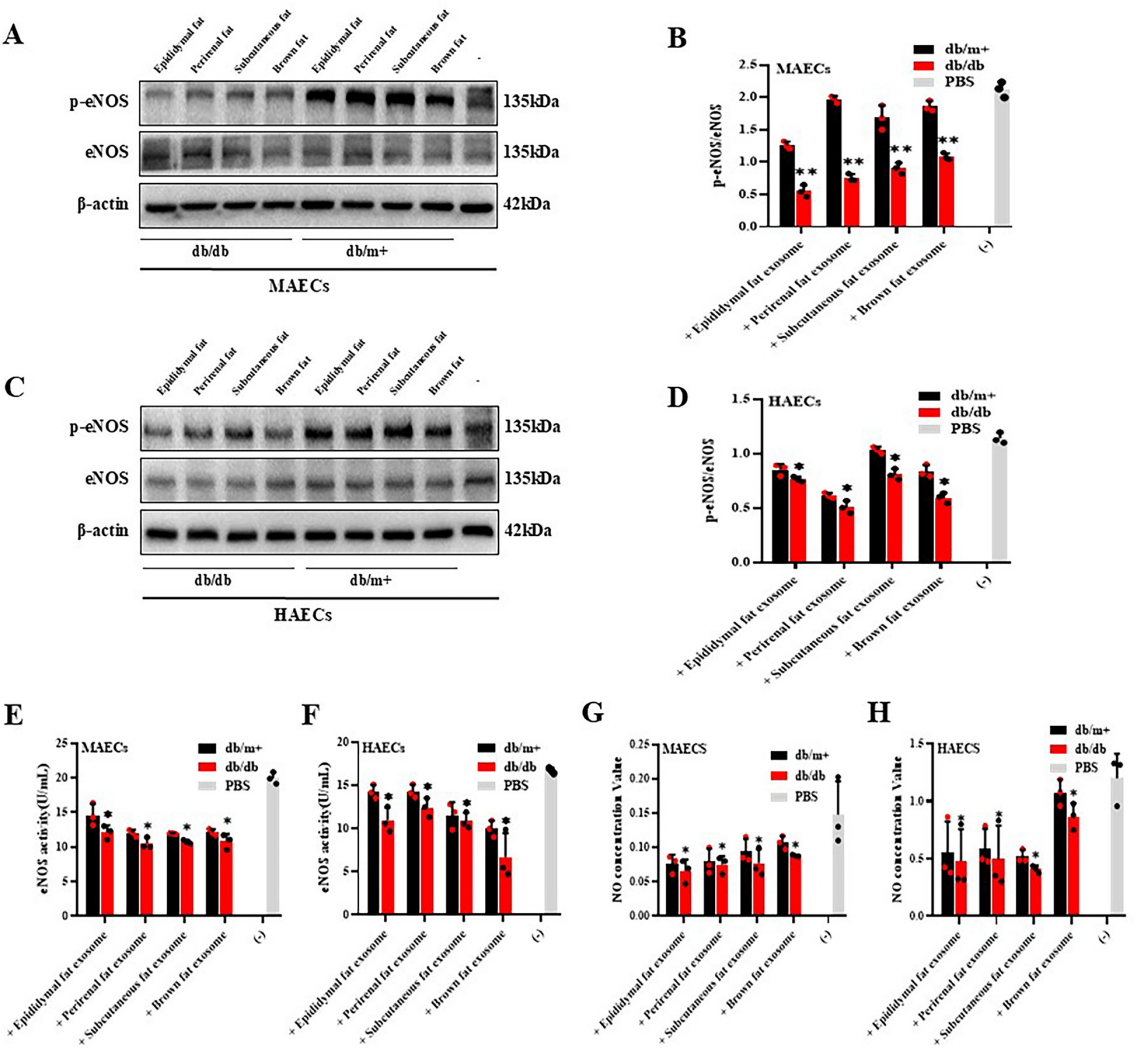
Cytological verification of vascular endothelial dysfunction caused by AT-exosomes. **(A–D)** The phosphorylation levels of eNOS in vascular endothelial cells were detected after the collection of AT-exosomes (*db/db* and *db/m+*) and co-culture with MAECs and HAECs. The changes in eNOS phosphorylation levels were different, *p < 0.05 and * *p < 0.01, *n* = 3. **(E–H)** The changes of eNOS activity and NO secretion in MAECs and HAECs were tested. The differences between the experimental group and the control group (PBS) were showed by *p < 0.05 and * *p < 0.01, *n* = 3.

Exosomes from epididymal, subcutaneous, and perirenal fat in diabetic mice had minimal impact on eNOS activity and phosphorylation in MAECs and HAECs, BAT-derived exosomes from diabetic mice steadily reduced both eNOS activity and phosphorylation levels in these cells (Figures 3A–F). Moreover, NO production was significantly diminished in cells co-cultured with BAT-derived exosomes from diabetic mice, while no significant effect（*p < 0.05 and **p < 0.01）on NO production was observed with perirenal AT-Exosomes (Figures 3G,H). These results indicated that BAT-derived exosomes from diabetic mice exert a more significant inhibitory effect on eNOS activity and NO production in aortic endothelial cells, positioning as a potential target for therapeutic intervention in diabetes-associated atherosclerosis.

### BAT-derived exosomes downregulate ITPR3 expression and impair Ca^2+^-dependent CAMKII phosphorylation in MAECs

**Figure 4:**
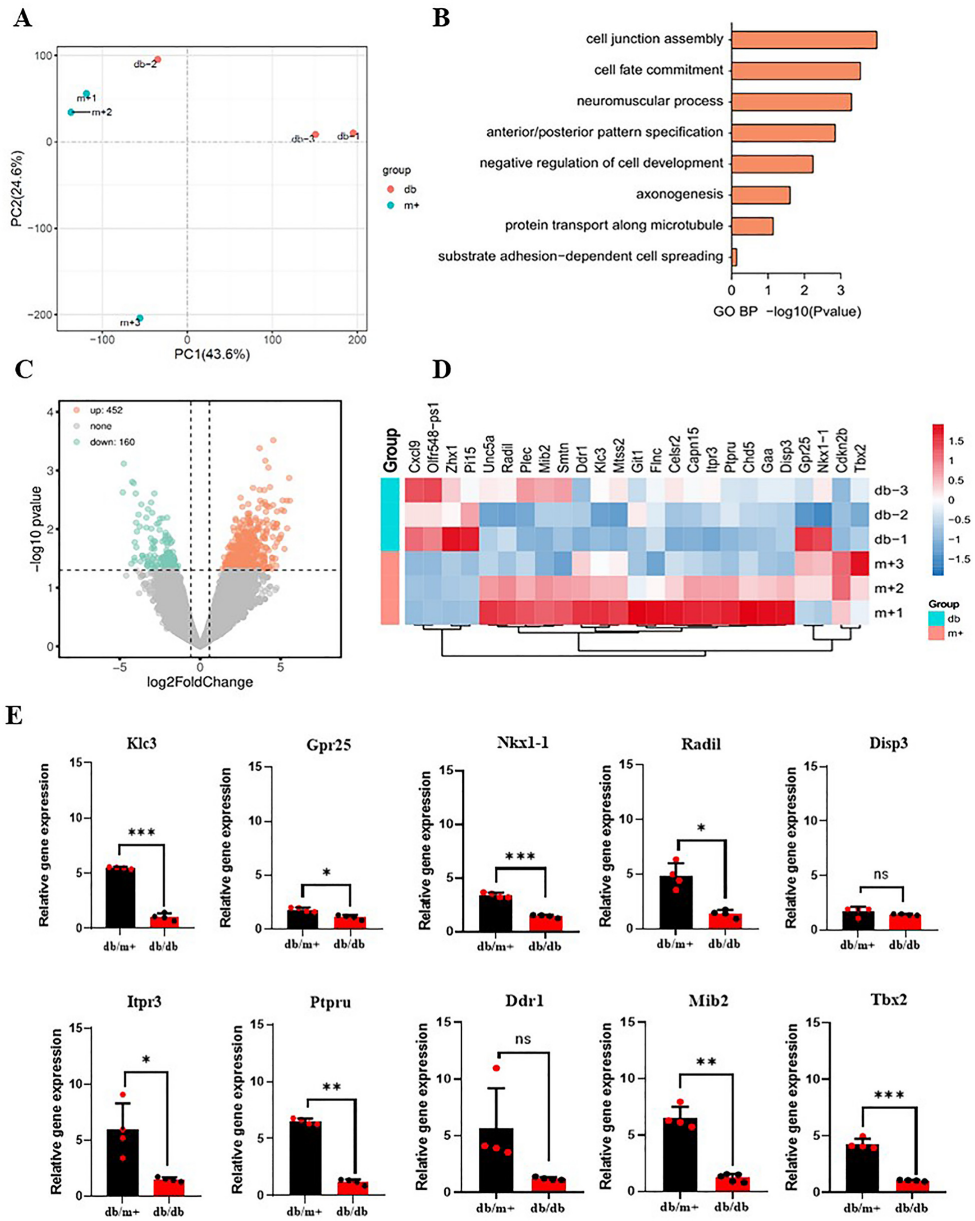
Gene sequencing of MAECs co-cultured with BAT-derived exosomes from diabetic mice and non-diabetic mice. **(A–D)** AT-exosomes from diabetic mice and non-diabetic mice served as controls to compare differential genes in MAECs. **(E)** Genes associated with cell proliferation were selected and qPCR was used to verify whether there were differences in the expression levels of target genes. *p < 0.05, ***p < 0.001, *n* ≥ 3.

MAECS co-cultured with BAT-derived exosomes from diabetic and non-diabetic mice were collected to sequence the RNA. RNA sequencing showed a predominant downregulation of genes in the MAECs exposed to BAT-derived exosomes from diabetic mice, compared to those from non-diabetic controls. Although some upregulated gene fragments were detected, their precise locations and identities remain uncharacterized (Figures 4A–C). Among the downregulated genes, several are associated with cell proliferation and angiogenesis. Ten genes related to these processes were selected for further validation by qPCR, and the results were consistent with the sequencing data. Notably, the expression of the ITPR3 gene was significantly reduced in MAECs co-cultured with BAT-derived exosomes from diabetic mice compared to those from non-diabetic mice (Figure 4D). The ITPR3 gene encodes the inositol 1,4, 5-triphosphate (IP3) receptor, a key regulator of intracellular Ca^2+^ release, which is localized to the endoplasmic reticulum and mediates the mobilization of Ca^2+^ stores (GeneCards). Western-blot analysis further showed that the phosphorylation trends of CAMKII, influenced by adipose-derived exosomes, aligned with changes in the ITPR3 expression (Figuures 5A–D). Notably, the Ca^2+^/CAMKII/eNOS/NO signaling axis is a critical pathway for endothelial function (19), and our findings highlight that reduced ITPR3 expression in diabetic conditions may impair this pathway, leading to diminished CAMKII activation, eNOS phosphorylation, and NO production in diabetes.

**Figure 5:**
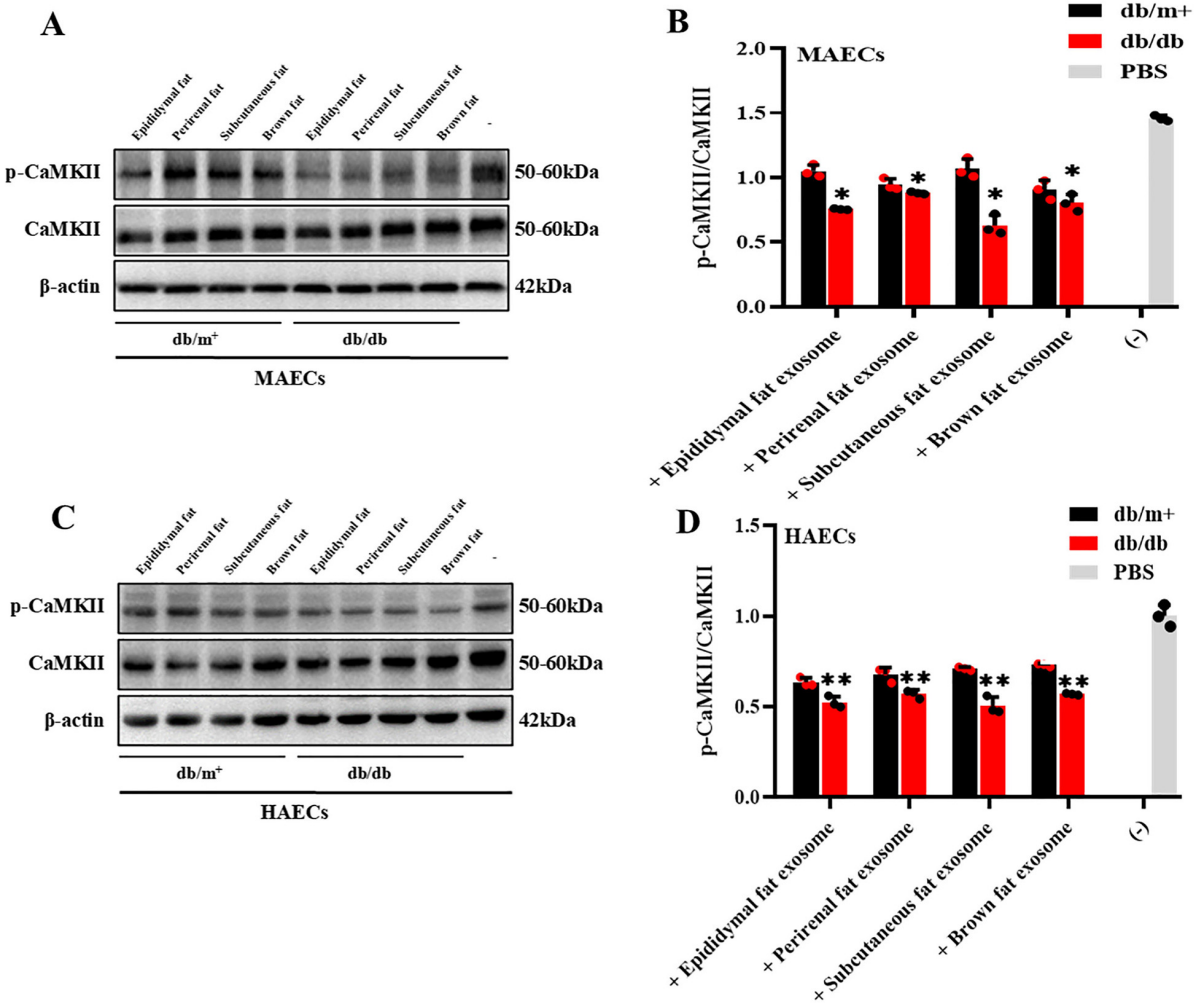
The expression levels of CAMKII phosphorylation in MAECs and HAECs were detected. **(A–D)** CAMKII phosphorylation level expressed different in MAECs and HAECs co-cultured with AT-exosomes, * *p < 0.01, *n* = 3.

### ITPR3 regulates the Ca^2+^/CAMKII/eNOS/No signaling axis

**Figure 6:**
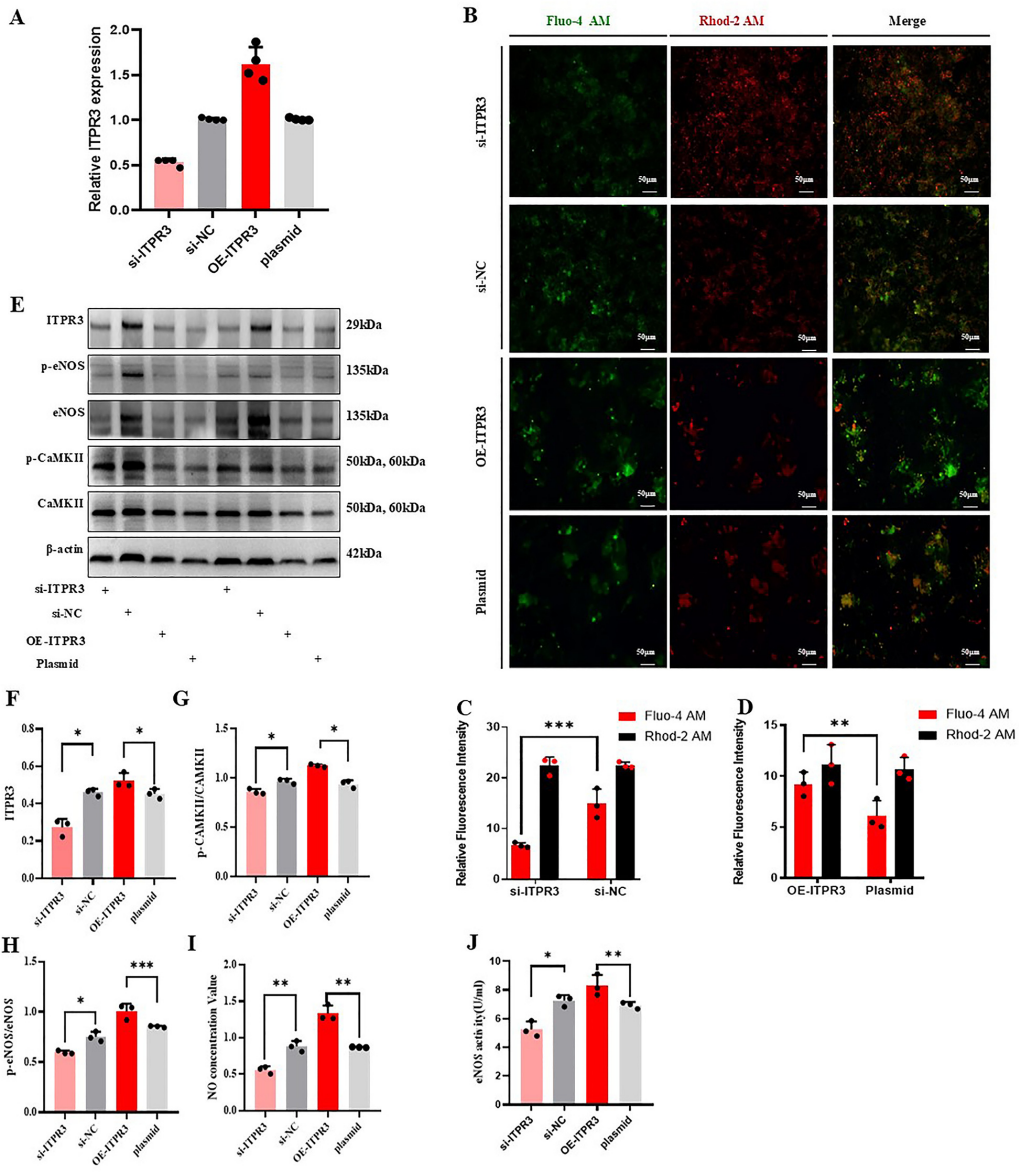
ITPR3 regulates Ca2+/CAMKII/eNOS/NO signaling pathways. **(A)** ITPR3 was verified whether down-regulated or up-regulated in MAECs, *n* ≥ 3. **(B–D)** The concentration of calcium ions in cytoplasm and mitochondria was stained with fluorescent dye, *n* ≥ 3. **(E–H)** The phosphorylation levels of eNOS and CAMKII in the cell model were detected. **(I, J)** The changes of NO secretion content and eNOS activity in the cell model were detected, *p < 0.05, **p < 0.01, ** *p < 0.001 indicates statistical significance, *n* ≥ 3.

To further evaluate the role of ITPR3 in regulating the Ca^2+^/CAMKII/eNOS/NO axis, we first confirmed the effective knockdown or overexpression of ITPR3 in MAECs through qPCR analysis. Changes in cytoplasmic and mitochondrial Ca^2+^ concentration were then assessed using Fluo-4 and Rhod-2 AM staining, respectively. The results showed that ITPR3 knockdown increased mitochondrial Ca^2+^ concentration without significantly affecting cytoplasmic Ca^2+^ levels. Conversely, in the ITPR3 overexpression model, cytoplasmic Ca^2+^ concentration increased, while mitochondrial Ca^2+^ remained unchanged (Figure 6B). Western-blot analysis corroborated these findings, showing that ITPR3 expression levels were positively correlated with eNOS and CAMKII phosphorylation (Figures 6A,C–F). Further, ELISA assay of eNOS activity revealed a positive correlation between reduced ITPR3 expression and decreased eNOS activity (Figure 6H), while NO production was also diminished in MAECs with lower ITPR3 levels (Figure 6G). These results collectively suggest that ITPR3 regulates the Ca^2+^/CAMKII/eNOS/NO signaling pathway, thereby regulating endothelium-dependent relaxation and contributing to vascular dysfunction.

## Discussion

This study investigates the effects of exosomes derived from various adipose tissue depots on vascular function in diabetic mice. Using ultracentrifugation, we successfully isolated high-purity exosomes from the culture medium of epididymal, subcutaneous, and BAT of both diabetic and non-diabetic mice. NTA confirmed that the exosomes had a similar size range (115.9–164.3 nm) across different adipose tissue, with no significant differences in size or concentration between the two groups. Western-blot analysis showed consistent expression of exosomal markers (CD81, CD63, and HSP70) across all samples, confirming the successful isolation of exosomes.

To explore the functional impact of these exosomes on vascular function, we cultured mouse aorta with AT-derived exosomes and assessed vascular contractility and relaxation function using a myograph. Epididymal, subcutaneous, and BAT-derived exosomes from diabetic mice significantly impaired aortic vasorelaxation compared to those from non-diabetic mice. This dysfunction was restored with exogenous NO supplementation, suggesting that adipose-derived exosomes can induce endothelial-dependent vasodilation impairment in diabetes. It showed that, WAT-exosomes and BAT-exosomes can cause the endothelial cells’ injure, which consistented with the conclusion in the previous literature that the pathological environment could make exosomes secreted by tissues have pathogenic effects (20).

Further validation through *in vitro* endothelial cell culture revealed that exosomes from diabetic mice suppressed eNOS activity, and decreased NO production in in both MAECs and HAECs. Notably, brown adipose-derived exosomes from diabetic mice exhibited the most significant and stable effects on endothelial dysfunction. Previous studies have highlighted the favorable proliferation and reparative properties of brown adipose. However, their role in the pathological context of diabetes appears to differ (6). We hypothesize that it is due to alterations in the underlying pathological conditions, which may modulate the functionality of BAT-derived exosomes. Specifically, when internalized by vascular endothelial cells, BAT-derived exosomes may impair endothelium-dependent vasodilation (14). To investigate this, we performed gene sequencing analysis on MAECs co-cultured with BAT-derived exosomes from both diabetic and non-diabetic mice, aiming to identify potential target genes.

RNA sequencing of MAECs revealed significant differences in gene expression between BAT-derived exosomes treatment groups from diabetic and wild-type mice. Genes down-regulated in BAT-derived exosomes treatment groups, particularly those involved in cell proliferation, were further validated through a comparative analysis with the GeneCards database, and their expression in diabetic mouse AT-exosomes was confirmed. Among them, we identified the ITPR3 gene, which encodes inositol 1,4,5-trisphosphate (IP3) receptor located on the inner surface of the cell membrane, including the endoplasmic reticulum. The receptor plays a key role in regulating the release of intracellular Ca^2+^ from the endoplasmic reticulum (21, 22). This suggested that BAT-derived exosomes mediated endothelial dysfunction may be linked to impaired Ca^2+^ signaling.

To further investigate this, we focused on the Ca^2+^/CAMKII/eNOS/NO signaling axis, a critical pathway for endothelial function (23). Our results indicate that exosomes derived from brown adipose tissue (BAT) in diabetic mice downregulate the CAMKII/eNOS/NO signaling pathway in endothelial cells. This occurs through a reduction in ITPR3 expression, which leads to decreased mobilization of Ca2 + and a lower level of CAMKII/eNOS phosphorylation. Consequently, this results in diminished nitric oxide (NO) production and impaired endothelium-dependent relaxation. Using models with both ITPR3 knockdown and overexpression in mouse aortic endothelial cells (MAECs), we observed a correlation between ITPR3 expression levels and cytosolic Ca2 + concentration. The lack of highly specific fluorescent reagents to accurately determine the location of Ca^2+^ within the endoplasmic reticulum (ER) of vascular endothelial cells, coupled with the presence of the mitochondrial-associated ER membrane (MAM), which links Ca^2+^ flow from the ER to the mitochondria, presents a challenge in studying these processes. MAM-mediated calcium transfer can cause a reduction in mitochondrial membrane potential, triggering endothelial cell apoptosis and contributing to plaque rupture in atherosclerosis (24, 25). To address this, we stained both the cytosol and mitochondria with Ca^2+^-specific dyes to observe the effects of ITPR3 on intracellular Ca^2+^ dynamics. Our results showed that ITPR3 expression positively regulated cytosolic Ca^2+^ levels. When cytosolic Ca^2+^ concentration increased, more Ca^2+^ bound to CAMKII, thereby enhancing its phosphorylation, which in turn elevated the phosphorylation of eNOS, resulting in increased NO production. These findings suggest that, under diabetic conditions, exosomes secreted by brown adipose tissue can be internalized by vascular endothelial cells, reducing ITPR3 expression and impairing Ca^2+^ binding. This inhibition of CAMKII phosphorylation decreases eNOS activity and NO production, ultimately causing impaired endothelium-dependent vasodilation. This underscores the essential role of Ca2 + signaling in endothelial cell function and suggests that exosomes derived from brown adipose tissue (BAT) may have the ability to remotely influence Ca2 + channel activity. Future research should focus on developing strategies to restore Ca^2+^ balance within endothelial cells, which could provide potential therapeutic approaches for addressing the vascular complications associated with type 2 diabetes mellitus (T2DM) and atherosclerosis. Additionally, key genes regulating ITPR3 in BAT-derived exosomes will be examined in detail.

## Conclusions

T2DM is associated with macrovascular complications such as atherosclerosis, with endothelial dysfunction serving as an early indicator of disease progression. Identifying the underlying pathways and key genes driving endothelial dysfunction in T2DM is essential for developing targeted therapies to address atherosclerotic complications. In this study, we suggest that ITPR3 gene as a critical player in this process, as its altered expression in BAT-derived exosomes under diabetic conditions disrupt cytoplasmic Ca^2+^ homeostasis in vascular endothelial cells. This dysregulation is associated with the CAMKII/eNOS/NO signaling axis, reducing phosphorylation levels of CAMKII and eNOS, and promoting vascular endothelial dysfunction. These findings offer novel therapeutic insights for managing atherosclerotic complications in T2DM.

## Data Availability

The data that support the findings of this study are available from NCBI but restrictions apply to the availability of these data, which were used under license for the current study, and so are not publicly available. Data are however available from the authors upon reasonable request and with permission of NCBI.
